# Polymorphism of Bismuth Sesquioxide. I. Pure Bi_2_O_3_

**DOI:** 10.6028/jres.068A.019

**Published:** 1964-04-01

**Authors:** Ernest M. Levin, Robert S. Roth

## Abstract

Stability relationships of the four polymorphs of bismuth oxide have been determined by means of DTA and high-temperature x-ray studies. The stable low-temperature monoclinic form transforms to the stable cubic form at 730 ±5 °C, which then melts at 825 ± 5 °C. By controlled cooling, the metastable tetragonal phase and/or the metastable body-centered cubic (b.c.c.) phase appear at about 645 °C. Whereas b.c.c. can be preserved to room temperature, tetragonal will transform to monoclinic between 550 and 500 °C. Tetragonal Bi_2_O_3_, however, is easily prepared by decomposing bismutite (Bi_2_O_3_·CO_2_) at 400 °C for several hours. The greatest transition expansion occurs at the monoclinic to cubic inversion, and cubic Bi_2_O_3_ shows the greatest coefficient of volume expansion. With exposure to air, Bi_2_O_3_ carbonates and partially transforms to bismutite and an unknown phase.

## 1. Introduction

Bismuth oxide is assuming an increasing importance in the ceramic field, particularly in the glass and electronics industries. A literature survey of the polymorphism of Bi_2_O_3_, in connection with phase studies of the systems Bi_2_O_3_—B_2_O_3_[1]^1^ and Bi_2_O_3_—Nb_2_O_5_[2], revealed unexplained inconsistencies in the data and no unequivocal interpretation. Furthermore, it was realized that a conclusive study of the polymorphic relationships would involve an identification of phases at temperature.

A number of investigators [3–10] have reported on temperatures of transitions and phase changes in Bi_2_O_3_. Schumb and Rittner [6] have reviewed the work prior to 1943. Table 1 summarizes the literature data through the work of the present authors, and includes, when given, methods of preparation and study, stable and unstable polymorphs, and temperatures of transitions.

It can be seen from the table that both pure and impure forms of Bi_2_O_3_ have been reported, with disagreement among investigators both as regards the identity and the stability of the pure phases. The essential conclusions which may be drawn from the previously reported data, as given in table 1, are as follows:

1. All investigators agree that monoclinic (Mon), pseudo-orthorhombic, (*α*-Bi_2_O_3_) is the stable low temperature form.
2. Schumb and Rittner believe that the tetragonal (Tet), or pseudocubic form (*β*-Bi_2_O_3_) is stable above 710 °C. Sillén suggested that this form might be simple cubic at higher temperatures. He based his hypothesis, in part, on analogy with cubic (C) As_2_O_3_ and Sb_2_O_3_.
3. Schumb and Rittner discovered a b.c.c. phase (*γ*-Hi_2_O_3_) representing a metastable form of Bi_2_O_3_. The finding was confirmed later by Auri- villius and Sillén, who agreed that the unit cell probably contained Bi_26_O_39_.
4. Gattow and Schröder, from the results of high-temperature x-ray, differential thermal analysis, and linear thermal expansion studies have found only two polymorphs of Bi_2_O_3_, namely the low temperature Mon form which transforms reversibly to a high temperature, face-centered cubic form, designated *δ*-Bi_2_O_3_.
5. A number of investigators have reported an impure b.c.c. phase, formed by contamination with porcelain, Si, Fe, Al, and other impurities. Sillén suggested the ideal formula type Me_2_Bi_24_O_40_.
6. Gattow and Schröder^2^ propose the designation δ*, β*, γ* for the impurity forms of C-, Tet-, and b.c.c.Bi_2_O_3_, respectively.
7. Previous investigators, usually using a cooling curve method, have reported transition temperatures between 700 and 717 °C. Liquidus temperatures vary from 817 to 825 °C.

The objective of this work (part I) was to clarify the polymorphic relationships of pure Bi_2_O_3_. This could best be accomplished by the method of high- temperature x-ray diffractometry, which avoids the problem of nonquenchable phases. The study was enlarged (part II) to include the effect of oxide additions on the polymorphism of Bi_2_O_3_, when a modification of the x-ray furnace permitted the determination of solidus temperatures and even, under favorable conditions, of liquidus temperatures. It was expected that the latter study would provide information on the impure forms.

## 2. Starting Materials

Two commercially available grades of bismuth oxide were used. One sample was of reported spectrographic purity; the other sample was certified reagent grade (ACS). The results of a qualitative spectrochemical analysis by the Analytical and Inorganic Chemistry Division of the National Bureau of Standards showed that the reagent grade sample contained iron and silicon (0.01 to 0.001%) in significantly higher concentrations than the spectrographic grade (less than 0.001%). Minor differences between the two samples were observed for a number of other elements (Ag, Al, Ca, Cr, Cu, Mg, Mn, Na, Ni, and Pb), which were each estimated to be present at less than 10 ppm metal in Bi_2_O_3_.

The following commercially available compounds were used for studying decomposition effects on the polymorphism of Bi_2_O_3_: bismutite (Bi_2_O_3_·CO_2_), bismuth hydroxide, bismuth nitrate (Bi(NO_3_)_3_·5H_2_O), bismuth subnitrate, bismuth subgallate, and bismuth subsalicylate.

## 3. Apparatus and Method

Polymorphic relationships in Bi_2_O_3_ were first deduced from differential thermal analyses of approximately 0.3 g samples. A few experiments on larger samples (approximately 3 g) gave no significant differences. The deduced relationships were then confirmed with the aid of the high-temperature x-ray diffractometer furnace. The diffraction data were obtained with a modification of the diffractometer furnace described by Mauer and Bolz [11]. The essential feature of the modification [12] involved a platinum sample holder to which the powdered samples could be made to adhere by using a thin smear of petroleum jelly. The petroleum jelly volatilizes at a low temperature, leaving the sample adhered to the surface.

It may be noted that the standard quenching technique was of limited value in this study, because the high-temperature form of Bi_2_O_3_ could not be quenched. However, in experiments dealing with the preservation at room temperature of the Tet and b.c.c. metastable phases a small quench furnace of low heat capacity was used. The furnace enabled one to control the cooling rate between two temperatures prior to quenching.

## 4. Results and Discussion

### 4.1. DTA and High-Temperature X-Ray Results

**Figure 1:**
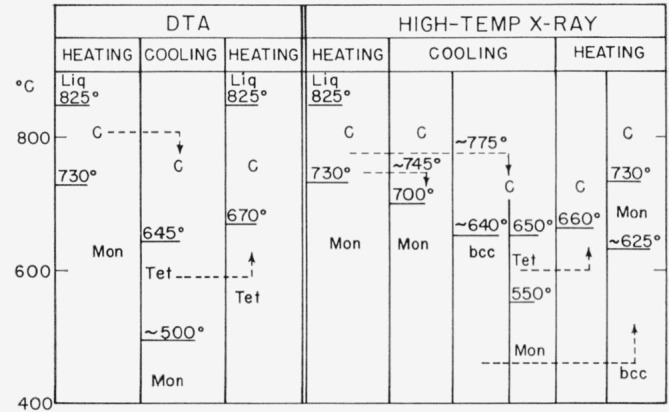
Schematic representation of results of differential thermal analysis (DTA) and high-temperature x-ray experiments on *Bi_2_O_3_* Dashed arrows indicate changes in direction of the heating and cooling schedule. Polymorphs: Mon—monoclinic, C—cubic, Tet—tetragonal, b.c.c.—body-centered cubic.

The results of differential thermal analyses of small samples (0.3 g) are summarized in figure 1. Phase identification was verified by comparison with the results obtained from the high-temperature x-ray diffraction experiments, also shown in figure 1.

The basic phase equilibrium relationships are revealed. At 730 ± 5 °C, the low temperature Mon form transforms to cubic Bi_2_O_3_ (C),^3^ which remains stable up to the melting point, 825 ± 5 °C. If the sample is not heated above about 745 °C (see high- temperature x-ray results), or is heated very rapidly to higher temperatures, then, on cooling, inversion to the Mon form takes place at 700 °C. If some of the Mon form persists above the transition, it will nucleate the inversion, on cooling.

If the C form is heated above about 775 °C, considerable supercooling of the C phase occurs, and tetragonal Bi_2_O_3_ forms at 650 to 645 °C. The Tet phase formed in this manner cannot be preserved to room temperature but transforms to the Mon phase at temperatures varying between 550 and 450 °C. If, however, the Tet phase is heated, conversion to the cubic form occurs between 660 and 670 °C.

Data from the high-temperature x-ray experiments revealed the b.c.c. phase in addition to the Tet phase. The b.c.c. phase appeared at a temperature slightly below that at which the Tet phase formed, and on reheating, the b.c.c. to C transition occurred slightly below that of the Tet to C transition. Once formed the b.c.c. phase could be preserved to room temperature by “furnace cooling.” Slow heating of the b.c.c. phase from room temperature yielded the stable Mon phase at about 625 °C.

A weight-loss determination on 0.3 g of the ACS grade Bi_2_O_3_ was made simultaneously with a DTA experiment (DTGA) on an independent sample. No detectable change in weight (within 0.001 g) was observed during heating and cooling through the various inversion temperatures.

**Figure 2:**
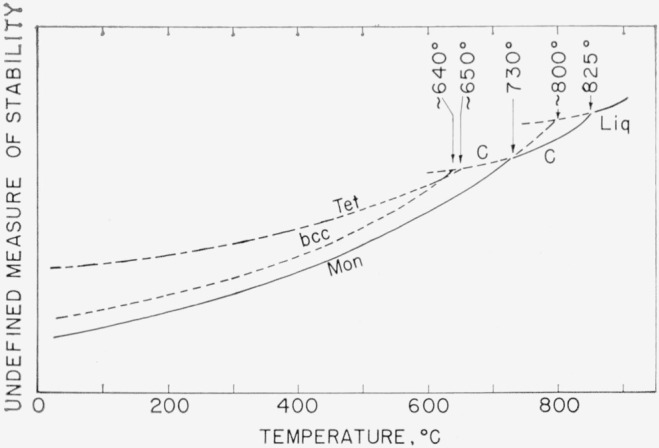
Postulated stability diagram of *Bi_2_O_3_* Polymorphs: Mon—monoclinic, C—cubic, Tet—tetragonal, b.c.c.—body centered cubic, Liq—liquid.

Figure 2 is a postulated stability diagram for the polymorphs of Bi_2_O_3_, based on the data in figure 1 and supported by additional experiments with the low-heat capacity quench furnace, to be discussed later. The ordinate in figure 2 is an undefined measure of stability, for example, free energy of formation or vapor pressure. The metastable melting point of Mon Bi_2_O_3_ is shown at approximately 800 °C, the temperature at which liquid was detected in the high-temperature x-ray furnace during rapid heating of the Mon form.

In the high-temperature x-ray experiments, the Tet phase appeared first on cooling of the C phase and disappeared last when transformation to the C form occurred. Therefore, in figure 2, the metastable C to Tet inversion is shown at a slightly higher temperature than the metastable C to b.c.c. inversion. However, considerable overlapping of the two forms occurred, indicating that the two inversion temperatures are near to each other. Below about 640 °C the b.c.c. phase is shown as more stable than the tetragonal phase, because only the former could be preserved to room temperature by supercooling from high temperatures.

**Figure 3:**
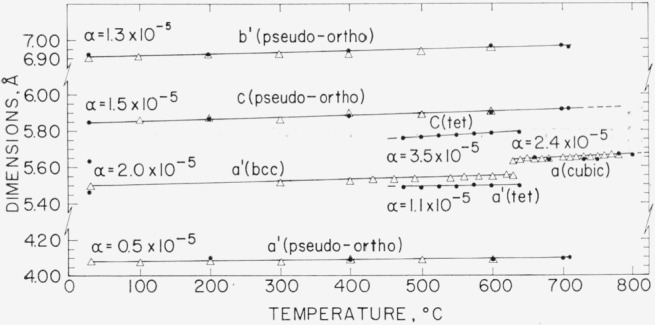
Pseudocell dimensions (based on Z = *2*) of the polymorphs of *Bi_2_O_3_* versus temperature Cell dimensions have been reduced to correspond to Z=2Bi_2_O_3_, in the following arbitrary manner: pseudo-orthorhombic—monoclinic polymorph *a*′(*Z*=2) = (½)*a*(*Z*=8), *b*′(*Z*=2) = (½)*b*(*Z*=8), *c*(*Z*=2) =*c*(*Z*=8). Cubic—cubic polymorph *a*(*Z*=2)=*a*(*Z*=2). Tet—tetragonal polymorph *a*′(*Z*=2) = (½)*a*(*Z*=8), *c*(*Z*=2)=*c*(*Z*=8). b.c.c.—body-centered cubic polymorph $a^{\prime }\left(Z=2\right)=\sqrt[{3}]{2/13}$ (*Z*=13). *α*=coefficient of linear thermal expansion, calculated by method of least squares on original data. ●—from certified reagent grade (ACS) Bi_2_O_3_. △—from “Spec. pure” grade Bi_2_O_3_.

Figure 3 shows a plot of pseudocell dimensions of the various polymorphs of Bi_2_O_3_ versus temperature. The data were obtained by use of the high-temperature x-ray furnace. For comparison purposes, the cell dimensions were reduced to correspond to a pseudounit cell volume for two molecules of Bi_2_O_3_, according to the arbitrary manner given in the caption of figure 3. The coefficient *α* of linear thermal expansion for each cell direction was calculated on the original unit cell data by applying least squares to the several sets of data. The cell dimension data were obtained for relatively low 2*θ* values (below 50°) ; and, consequently, the accuracy of the thermal expansion values is estimated to be in the order of ±0.2 × 10^−5^.

The pseudo-orthorhombic (Mon) form showed low linear thermal expansion coefficients, namely, 0.5 × 10^−5^, 1.3 × 10^−5^, and 1.5 × 10^−5^ Å/Å/deg for the *a, b*, and *c* directions, respectively. The average of these three x-ray determined values is 1.1 × 10^−5^/deg, in fair agreement with the values reported by Gattow and Schröder [10], using a dilatometric method. Their values ranged from 1.22 × 10^−5^/deg at 100 to 200 °C to 1.48 × 10^−5^/deg at 575 to 675 °C. The coefficient of linear thermal expansion found in the present work for the C form, 2.4 × 10^−5^/deg, is almost half that reported by Gattow and Schröder, 4.36 × 10^−5^/deg. The *c* axis for Tet Bi_2_O_3_ showed the largest coefficient of linear thermal expansion, 3.5 × 10^−5^/deg. Based on volume expansion, however, the C form would show the greatest coefficient of expansion (7.2 × 10^−5^/deg), and the Mon form, the lowest expansion (3.3 × 10^−5^/deg).

**Figure 4:**
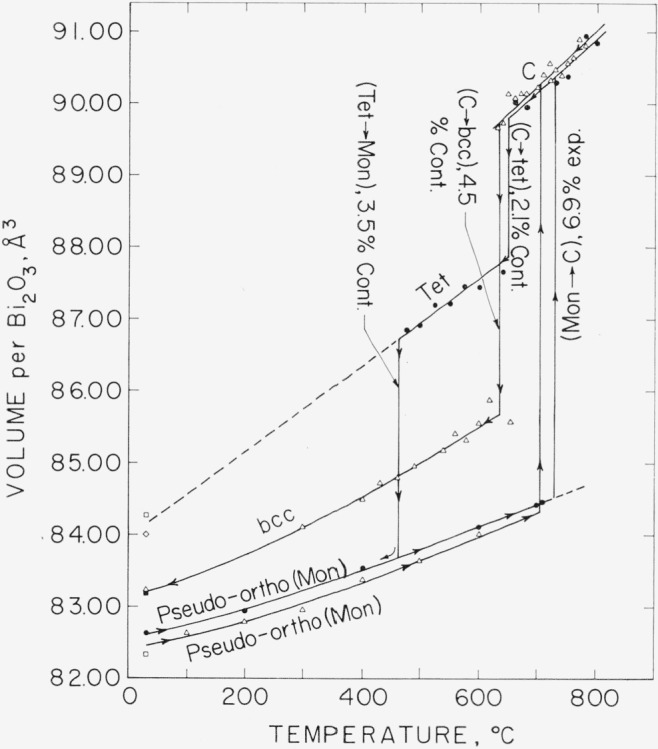
Volume per molecule versus temperature for the polymorphs of *Bi_2_O_3_* (Calculated from the unit cell dimensions obtained with the high-temperature x-ray diffractometer furnace). Polymorphs: Pseudo-orthorhombic (Mon)—monoclinic, C—cubic, Tet—tetragonal, b.c.c.—body-centered cubic. % exp.—percentage expansion at transition. % cont.—percentage contraction at transition. ●—from certified reagent grade (ACS) Bi_2_O_3_. △—from “spec pure” grade Bi_2_O_3_. ◊—from decomposition of bismutite. □—data taken from Sillén [5]. ■—data taken from Schumb and Rittner [6].

Because the different polymorphs of Bi_2_O_3_ do not have the same number of molecules per unit cell, a comparison of unit cell dimensions is not as instructive as a volume comparison. Figure 4 shows a plot of temperature versus the volumes per molecule of the different polymorphs of Bi_2_O_3_. The percentage expansion or contraction at a phase transition is also given.

It may be seen that the Mon phase shows a large expansion, 6.9 percent, when transforming to the C phase at 730 °C. Gattow and Schröder reported a volume expansion at the transition temperature of 4.11 ±0.06 percent. Thus the present authors obtained a higher percentage of volume expansion at the Mon to C transition temperature and a lower coefficient of linear thermal expansion for the cubic form, than did Gattow and Schröder. Gattow and Schröder used a relatively fast heating rate of 7°/min, but the Mon to C transition is not instantaneous and occurs over a temperature range. Gattow and Schroder’s results may be explained on the basis that in their experiments all of the Mon form did not transform at the inversion temperature but continued to transform during heating of the C phase. Such a situation would lead to an apparently low Mon to C expansion and an apparently high thermal expansion of the C phase.

The C to Tet transition involves a relatively small contraction, 2.1 percent, indicating the similarity in unit cell volumes of the two forms. The contraction in the transition from the C to b.c.c. is 4.5 percent. It is seen that the temperature dependence of the volume per Bi_2_O_3_ varies for the polymorphs and that the room temperature volume differences are considerably less than at higher temperatures.

### 4.2. Low-Heat Capacity Furnace Results

The C form of Bi_2_O_3_ cannot be “quenched” at room temperature. In previous phase equilibrium studies by the present investigators [1, 2] only the Mon phase was obtained in pure Bi_2_O_3_ by quenching samples in sealed platinum tubes from all temperatures up to 100 °C above the melting point of Bi_2_O_3_. The stability diagram (fig. 2), however, indicated a method whereby the metastable phases might be obtained at room temperature, as follows: (a) Heating a sample in a sealed platinum tube to a temperature at which it would be definitely all cubic (above about 775 °C) or liquid, (b) Slow cooling of the sample to about 625 °C, with the possibility that the C phase would be supercooled below 730 °C and would transform to one of the metastable phases, (c) Quenching the metastable phase existing at temperature.

Accordingly, a number of experiments were performed with the low heat capacity furnace in which temperatures and rates of cooling were varied. Identification was by means of the x-ray powder diffraction method applied to the quenched samples. The essential results are summarized as follows:

- Pure b.c.c. Bi_2_O_3_ was formed by heating a sample above the liquidus, at 850 °C for 10 min, cooling to 625° C in about 45 min, and continued heating at this temperature for 5 min, before quenching.
- Body-centered cubic Bi_2_O_3_ with a trace of Tet Bi_2_O_3_ could be formed by heating a sample below the liquidus, to 775 °C. The cooling schedule was similar to that in (a).
- Pure b.c.c. Bi_2_O_3_ could be formed readily below the liquidus temperature by a recycling heat treatment, namely, by heating twice to 780 to 785 °C and cooling twice to about 625 °C, before quenching.
- These experiments never yielded a large amount of Tet Bi_2_O_3_. In several cases, however, the Mon phase was found with a trace of the Tet; it seems probable that at temperature the specimens were Tet.

The work of Royen and Swars [8] is related to the problem of forming Tet Bi_2_O_3_ from pure bismuth oxide. They formed some Tet Bi_2_O_3_ by fast quenching of molten Bi_2_O_3_ from temperatures above about 850 °C. Their work was essentially substantiated in the present investigation, by quenching specimens enclosed in platinum foil envelopes from temperatures of 930 °C (5 min heating period) and 980 °C (1 min heating period). It may be noted that although the Tet phase was present in appreciable amounts, the major phase consisted of Mon Bi_2_O_3_. Quenching from the higher temperature did not yield an appreciable higher percentage of the Tet phase. However, samples contained in sealed platinum tubes and quenched from the same temperatures showed only Mon Bi_2_O_3_.

### 4.3. Aging Experiments on Bi_2_O_3_

During the course of the work, an aging effect on Bi_2_O_3_ was discovered. Material from a freshly opened bottle of Bi_2_O_3_ gave an x-ray powder pattern for pure Mon Bi_2_O_3_. About six months later, however, material from the same bottle, which had not been desiccated, showed the prominent diffraction peaks corresponding to bismutite (Bi_2_O_3_·CO_2_). The effect of the bismutite formation seemed to be a tendency toward lowering of the temperature of the Mon to C inversion to about 715 °C and also toward increasing the probability of forming the b.c.c. phase during supercooling of cubic Bi_2_O_3_. This effect may be seen in figure 4, for a sample of spectrographic purity and offers a possible explanation for the lower inversion temperature reported by previous investigators (see table 1). It should be emphasized, however, that the b.c.c. phase could be formed from pure material, as discussed previously under 4.1 and 4.2, above.

This chance discovery of an aging effect led to a number of experiments (summarized in table 2) in an attempt to accelerate the process. Experiments in which pure Bi_2_O_3_ was suspended over water in a stoppered flask and to which additions of dry ice were made periodically produced bismutite in small to moderate amounts, after 17 days. At the end of 55 days there was no increase in amount of bismutite formed. After 76 days, samples exposed to water vapor and CO_2_ or to air at room temperature showed a large amount of an unidentified phase (X in table 2). With continued exposure the unidentified phase, in both experiments, increased at the expense of the bismutite and Mon phases. Attempts to rapidly accelerate the formation of bismutite or the unknown phase by passing over the heated sample a stream of (1) CO_2_ gas, (2) CO_2_ saturated water vapor, or (3) water saturated air, were unsuccessful (see last 3 experiments of table 2). The aging experiments demonstrate the fact that Bi_2_O_3_ is reactive under atmospheric conditions and must be tightly sealed and desiccated to avoid compositional changes.

### 4.4. Thermal Decomposition Experiments

The discovery of bismutite (Bi_2_O_3_·CO_2_) in aged bismuth oxide samples led directly to thermal decomposition studies of bismutite and other bismuth containing organic compounds (see table 3). Approximately 1 g samples were accurately weighed into 30 ml platinum crucibles, heated in an electric resistance furnace at successively higher temperatures, and cooled in air. Ignition losses and x-ray powder diffraction patterns of the samples were obtained for the successive heat treatments.

It is seen from table 3 that bismutite heated at 390 °C for 66 hr or at 400 °C for 18 hr produced pure Tet Bi_2_O_3_. Within the limit of accuracy of the weight loss measurements, it appears that all of the CO_2_ was driven off. At 490 °C, the Tet Bi_2_O_3_ was transformed to the stable Mon phase. Rapid cooling of a sample from 800 °C, by immersion of the bottom of the crucible in cold water, produced the b.c.c. phase.

Bismuth subsalicylate and bismuth subgallate when heated at 300 °C for 16 hr also yielded pure Tet Bi_2_O_3_. At the 400 °C heat treatment, the Tet phase was converted to the Mon phase.

The bismuth hydroxide sample, which was shown by x-ray analysis to be mainly bismutite, yielded three forms of Bi_2_O_3_ during decomposition. A large amount of the tetragonal phase was formed at 400 °C. At 450 and 500 °C, the Tet phase had disappeared, and the sample contained a large amount of the b.c.c. phase plus a small amount of the Mon phase. This transition supports the previously drawn conclusion that, at lower temperatures, the b.c.c. phase is more stable than the Tet phase. The 600 °C heat treatment produced an increasing amount of the Mon phase, at the expense of the b.c.c. phase. The remaining b.c.c. phase showed a shift in diffraction peaks corresponding to smaller *d* values. This shift would be consistent with a decrease in unit cell size, when certain foreign ions are added to pure Bi_2_O_3_, as will be discussed in part II.

Bismuth nitrate and bismuth subnitrate yielded some Tet Bi_2_O_3_ during decomposition, but never in major amount. By the end of the 500 °C heat treatment the samples were all Mon Bi_2_O_3_. Bismuth oxyhydroxide transformed directly from the amorphous state to the Mon form of Bi_2_O_3_.

These experiments indicate that the decomposition of organic bismuth compounds at temperatures below 400 °C tend to produce the tetragonal form of Bi_2_O_3_. If, however, higher temperatures are required for complete decomposition, then the stable Mon Bi_2_O_3_ phase is formed.

## 5. Summary

By means of DTA and high-temperature x-ray studies it has been shown that pure bismuth oxide possesses two stable and two metastable polymorphs. A stable reversible transition between the low temperature Mon and the high temperature cubic form occurs at 730 *±* 5 °C. The Mon to cubic transition is accompanied by a volume expansion of 6.9 percent. The cubic form shows the greatest coefficient of volume expansion (7.2 × 10^−5^/deg). The melting point of the cubic phase is 825 ± 5 °C. Supercooling of the cubic form below the 730 °C Mon transition produces either the metastable tet form at 650 °C or the metastable b.c.c. form at about 640 °C. The results are given in table 1. Whereas by fast cooling, the b.c.c. phase can be preserved to room temperature, the tetragonal phase transforms to the stable Mon phase between 550 and 450 °C. Tetragonal bismuth oxide, however, can be formed readily by the decomposition of bismutite at 400 °C for 18 hr or by the decomposition of bismuth subgallate and bismuth subsalicylate at 300 °C for 16 hr. The Tet bismuth oxide so formed is preserved at room temperature. Bismuth oxide is subject to an aging effect in air and transforms partially to bismutite. With continued exposure an unidentified phase forms.

## Figures and Tables

**Table 1:** Literature summary on polymorphism of Bi_2_O_3_

Investigator	Form	Method of preparation	Notes	Method of study	Transition temp.	Melting temp.
						
Guertler (1903) [3]		From basic nitrate in porcelain crucible.	(Contains 1.3% silic acid)	Cooling curve. Heating curve. Cooling curve.	704±1 °C 705±1 °C	820±2°C 825±5° 860°
Belladen (1922) [4]		Calcination of basic nitrate.		Cooling curve.		817°
Sillén (1937) [5]	*α*-Bi_2_O_3_ (Mon) *β*-Bi_2_O_3_ (Tet)	Bi(NO_3_)_3_ (aq)+ KOH (aq). Condensation of bismuth vapor from graphite furnace, in O_2_ stream.	Stable low-temp. form. May be cubic at higher temperatures. *a* = 10.93 *c* = 5.62 kx.			
	b. c. c. Cubic Bi_2_O_3_	Fused in porcelain at 900 °C for 5 min or with Al_2_O_3_ or Fe_2_O_3_. Fused in porcelain at 900 °C for 20 min.	Suggested formula type: Me_2_Bi_24_O_40._ *a* = 10.08 kx. Contaminated with silicon, *a* = 5.525 kx.			
Schumb and Rittner (1943) [6]	*α*-Bi_2_O_3_ (Mon) *β–*Bi_2_O_3_ (Tet) *γ*-Bi_2_O_3_ (b. c. c.) b. c. c. Cubic Bi_2_O_3_	Purification of C. P. Bi_2_O_3_ by heating at 750 °C and leaching. Arc between graphite electrode and molten bismuth in O_2_ stream. Controlled cooling of *β*-Bi_2_O_3_ from 750–800 °C in Pt crucible. Fusion (875 °C) of Bi_2_O_3_ in porcelain and with SiO_2_. Fusion (875 °C) of Bi_2_O_3_ in porcelain or silica crucible at 875° and water quenching.	99.99±0.06% Bi_2_O_3_. Stable below 700 °C. 99.8±0.2% Bi_2_O_3_. Stable above 710°. *a* = 10.93 *c* = 5.63 kx. Metastable; probably Bi_26_O_39_. *a* = 10.245 kx. Impure phase: *a* = 10.090 kx. Impure phase: *a*=5.525 kx.	X-ray examination of rapidly cooled Sample	710°	
Aurivillius and Sillén (1945) [7].	*γ*-Bi_2_O_3_ (b. c. c.)	Not given. Same as Schumb and Rittner (1943).	Stated to be pure Bi_2_O_3_. Probably Bi_26_O_39_. *a* = 10.243 kx.	Cooling curve of a large quantity in Pt crucible.	≈700°	
Royen and Swars (1957) [8].	*β*-Bi_2_O_3_ (Tet)	Quenched molten Bi_2_O_3_ from “850° or perhaps 920° C”.	*α*-Bi_2_O_3_ may also be present in minor amt.			
Belayev and Smolaynenov (1962) [9].		Bi_2_O_3_ calcined at 500 °C/1–1.5 hr.		Visual polythermal.	704°	819°
Gattow and Schröder (1962) [10].	*α*-Bi_2_O_3_ (Mon) *δ*-Bi_2_O_3_ (Cubic) δ*-Bi_2_O_3_ β*-Bi_2_O_3_ γ*-Bi_2_O_3_	Not given.	100.0_0_±0.10% Bi_2_O_3_. Stable low- temp. form. Stable high-temp. form. *a* = 5.66±0.008 A (at 750 °C). Cubic impurity form.^a^ Tetragonal impurity form.^b^ Body-centered cubic impurity form.^c^	DTA and high-temp. x ray. High-temp. x ray. X ray. X ray. X ray.	717±7 °C	824±2 °C
Levin and Roth (present work)^d^	Mon Bi_2_O_3_ Cubic Bi_2_O_3_ Tet Bi_2_O_3_^e^ b. c. c.^f^	Commercial “Spec. pure” and ACS Grades. …..do………………. ……do……………… ……do……………..	Stable low-temp. form. Stable high-temp. form. *a*= 5.66 Å (at 750°C). Metastable; formed from super-cooling Cubic Bi_2_O_3_. *a* = 10.93 Å *c* = 5.63 Å Metastable; formed from supercooling cubic Bi_2_O_3_. Anion impurities may be important. *a* = 10.268 Å.	DTA and high-temp. x ray. High-temp. x ray. DTA and high-temp. x ray. High-temp. x ray.	730±5 °C	825±5 °C

a Obtained by fast quenching of molten bismuth oxide mixtures heated at 800 to 1000 °C for 15 to 60 min. Bi_2_O_3_ mixed with: As_2_O_3_, Sb_2_O_3_, La_2_O_3_, Nd_2_O_3_, Cr_2_O_3_, SiO_2_, SnO_2_, TiO_2_, V_2_O_5_, Nb_2_O_5_, Ta_2_O_5_, WO_3_. The same phase was obtained from aqueous solution in the system Bi_2_O_3_–MoO_3_.

b Obtained under certain conditions with mixtures of Bi_2_O_3_ and: Al_2_O_3_, Ga_2_O_3_, In_2_O_3_, Y_2_O_3_, Tl_2_O_3_, Fe_2_O_3_, Cr_2_O_3_ PbO_2_, MnO_2_, CeO_2_, ZrO_2_, ThO_2_.

c Sillén’s body-centered cubic (b. c. c.) type. Impurity oxides not listed.

d See also [1, 2].

e Pure tetragonal Bi_2_O_3_ at room temperature can be obtained easily by heating Bi_2_O_3_·CO_2_ (bismutite) at about 400 °C for 2 to 16 hr.

f A related b. c. c. form is either stable or metastable depending on the cation impurity (discussed in part II).

**Table 2:** Aging experiments on *Bi_2_O_3_*

Sample grade	Environment		Time	X ray results^a^
	Atmosphere	Temp.		
				
		*°C*	*Days*	
ACS	Air	≈25	76	Mon (m)+Bi_2_O_3_·CO_2_ (m)+X^b^ (I)
“Spec pure”	Air	≈25	76	Mon (m)+Bi_2_O_3_·CO_2_ (s)+X^b^ (I)
ACS	Air	≈25	147	Mon (m)+Bi_2_O_3_·CO_2_ (tr)+X^b^ (I)
“Spec pure”	Air	≈25	147	Mon (m)+Bi_2_O_3_·CO_2_ (s−)+X^b^ (I)
ACS	H_2_O sat. air + CO_2_^c^	≈25	17	Mon (I)+Bi_2_O_3_·CO_2_ (s+)
“Spec pure”	H_2_O sat. air + CO_2_^c^	≈25	17	Mon (I)+Bi_2_O_3_·CO_2_ (m)
ACS	H_2_O sat. air + CO_2_^c^	≈25	55	Mon (I)+Bi_2_O_3_·CO_2_ (s)
“Spec pure”	H_2_O sat. air + CO_2_^c^	≈25	55	Mon (I)+Bi_2_O_3_·CO_2_ (m)
ACS	H_2_Osat. air + CO_2_^c^	≈25	76	Mon (I)+Bi_2_O_3_·CO_2_ (s−)+X^b^ (I)
“Spec pure”	H_2_O sat. air + CO_2_^c^	≈25	76	Mon (I)+Bi_2_O_3_·CO_2_(m)+X^b^ (m)
ACS	H_2_O sat. air	≈25	167	Mon (m)+Bi_2_O_3_·CO_2_ (tr)+X^b^ (I)
“Spec pure”	H_2_O sat. air	≈25	167	Mon (m)+Bi_2_O_3_·CO_2_ (s−)+X^b^ (m)
ACS	H_2_O sat. air^d^	140	1	Mon (I)
ACS	CO_2_^d^	157	1	Mon (I)
ACS	H_2_O sat. CO_2_^d^	145	2¾	Mon (I)

a Mon-monoclinic phase: 1—large percentage, m—moderate percentage, s—small percentage, tr—trace.

b Unknown phase: *d* values of 3.71 Å, 3.68 Å, 3.02 Å (1), 2.74 Å, 2.45 Å.

c Specimen in stoppered flask, suspended over water, with periodic additions of dry ice.

d Gas or vapor stream directed through heated chamber containing specimen.

**Table 3:** Thermal decomposition experiments on bismuth compounds

Chemical		Heat schedule		Total ignition Loss	X ray results^a^
Name	Approximate formula (theoretical ignition loss)	Temp.	Time		
					
		°*C*	*Hr*		
Bismutite (U.S.P.)	Bi_2_O_3_·CO_2_ (8.63%)	390	1.5	8.08	Tet Bi_2_O_3_
		390	66	8.65	Tet Bi_2_O_3_ *a*-10.9 Å, c-5.62 Å
		490	1.5	8.75	Mon Bi_2_O_3_
		800	1^b^	8.90	b.c.c. Bi_2_O_3_+? (tr)
Bismutite (U.S.P.)	Bi_2_O_3_·CO_2_ (8.63%)	200	16	0.29	Bi_2_O_3_·CO_2_
		300	18	1.05	Bi_2_O_3_·CO_2_
		350	4	5.47	Tet Bi_2_O_3_ (1)+Bi_2_O_3_·CO_2_
		400	18	9.08	Tet Bi_2_O_3_
		800	¼^b^		b.c.c. Bi_2_O_3_
		775	2		Mon Bi_2_O_3_
Bismuth subsalicylate (powder, U.S.P.)	Bi (C_7_H_5_O_3_)_3_Bi_2_O_3_ (35.6%)	137	16	0.06	Bismuth subsalicylate (?)
		200	16	.42	Subsalicylate (?)+unknown
		300	16	35.0	Tet Bi_2_O_3_
		400	16	35.54	Mon Bi_2_O_3_
		450	16	35.57	Mon Bi_2_O_3_
		500	16	35.57	
Bismuth subgallate (powder)	Bi (OH)_2_C_7_H_5_OH (43.4%)	137	16	10.10	Bismuth subgallate (?)^c^
		200	16	14.02	Unknown
		300	16	45.71	Tet Bi_2_O_3_
		400	16	45.91	Mon Bi_2_O_3_
		450	16	45.92	Mon Bi_2_O_3_
		500	16	45.92	…………………………….
Bismuth hydroxide^d^	Bi (OH)_3_ (10.4%)	137	16	0.13	Bi_2_O_3_·CO_2_ (I)+? (s)
		200	16	.28	Bi_2_O_3_·CO_2_ (I)+? (s)
		300	16	1.13	Bi_2_O_3_·CO_2_
		400	16	8.18	Tet Bi_2_O_3_ (I)+b.c.c. (s)
		450	16	8.42	b.c.c. Bi_2_O_3_ (I)+Mon Bi_2_O_3_ (s)
		500	16	8.46	b.c.c. Bi_2_O_3_ (I)+Mon Bi_2_O_3_ (s)
		600	2	8.50	Mon Bi_2_O_3_ (m)+b.c.c. Bi_2_O_3_ (m)^e^
		700	18	8.50	Mon Bi_2_O_3_ (I)+b.c.c. Bi_2_O_3_ (s)^e^
Bismuth nitrate (crystals, ACS)	Bi (NO_3_)_3_·5H_2_O (52.0%)	137	16	37.50	Unknown
		200	16	39.58	Unknown
		300	16	45.38	Amorphous (I)+Unknown (m)
		400	16	49.60	Unknown (I)+Tet Bl_2_O_3_ (m)
		450	16	51.44	Mon Bi_2_O_3_
		500	16	51.44	Mon Bi_2_O_3_
		800^f^	……….	……….	b.c.c. Bi_2_O_3_+? (tr)^f^
Bismuth subnitrate (powder)	Bi (OH)_2_NO_3_ (23.6%)	137	16	2.95	Amorphous + Unknown
		200	16	4.44	Amorphous + Unknown
		300	16	10.81	Amorphous + Unknown
		400	16	16.52	Intermediate Unknown
		450	16	19.36	Mon Bi_2_O_3_+Tet Bi_2_O_3_ (s)+Intermediate Unknown
		500	16	20.25	Mon Bi_2_O_3_
Bismuth oxyhydroxide^g^	BiO(OH) (3.72%)	135	16	.35	Amorphous+Unknown
		200	16	.95	Amorphous+Unknown
		250	16	1.57	Amorphous+Unknown
		300	17	1.71	Unknown+Mon Bi_2_O_3_ (s)
		350	7	1.74	Unknown+Mon Bi_2_O_3_ (m)
		400	16	2.77	Unknown+Mon Bi_2_O_3_ (m+)
		450	7	5.48	Mon Bi_2_O_3_
		500	16	5.62	Mon Bi_2_O_3_
		550	16	5.62	………………………

a Polymorphs: Mon-monoclinic, Tet-tetragonal, b.c.c.-body-centered cubic. Percentages: 1-large, m-moderate, s-small, tr-trace.

b Platinum crucible containing specimen removed from furnace at temperature and immediately chilled by dipping into ice water. (Water did not touch specimen.)

c Poorly crystalline.

d Commercially obtained preparation, essentially bismutite by x-ray analysis.

e *d* values show shift, indicating smaller unit cell.

f X ray analysis after a DTA experiment.

g Prepared in National Bureau of Standards Laboratory by Johan H. deGroot by adding bismuth nitrate to boiling 20 percent NaOH soln., in Pt dish. Material dried at 60 °C for 17 hr, then at 85 °C for 65 hr.

## Footnotes

1 Figures in brackets indicate the literature references at the end of this paper.

2 Gattow and Schröder [10] report a metastable bismuth oxide phase, Bi_3_O_5_, formed under oxidizing conditions and very similar in structure to Tet, *β*-Bi_2_O_3_; however, no identifying characteristics other than chemical analysis are given. Moreover, it transforms to *β*-Bi_2_O_3_ in about 3 months at 20 °C, or more rapidly at 470 ± 50 °C, or in a partial vacuum.

3 The x-ray diffraction powder data fit a face-centered cubic cell. Sillén has interpreted the cubic phase as being simple cubic, whereas Gattow and Schröder [10] consider the symmetry to be face-centered cubic.
